# Dataset on retail outlet product prices for Botswana, Lesotho and South Africa

**DOI:** 10.1016/j.dib.2018.05.006

**Published:** 2018-05-08

**Authors:** Mamello A. Nchake, Lawrence Edwards, Neil Rankin

**Affiliations:** a Department of Economics, National University of Lesotho and School of Economics, University of Cape Town, South Africa; b School of Economics, University of Cape Town, South Africa; c Department of Economics, University of Stellenbosch, South Africa

**Keywords:** Retail products, Consumer prices, Southern Africa

## Abstract

The data presented in this article are related to the research article entitled “Closer monetary union and product market integration in emerging economies: Evidence from the Common Monetary Area in Southern Africa” (M. A. Nchake, L. Edwards, N. Rankin, 2017) [1]. This article describes the monthly retail product prices used in the compilation of the consumer price index of Lesotho, South Africa and Botswana, and collected by the statistical offices in the respective countries. The data are provided at the product level and vary across cities and across time. Each individual product has information on the date (month and year), city, product and unit codes, units of measurements and, in some cases, brand name of that product. The data is made publicly available to enable replication analysis or to extend on the existing results.

**Specifications Table**

**Table:** 

**Subject area**	International economics, Private Enterprise Development
**More specific subject area**	Retail firms and micro price data
**Type of data**	Tables and metric variables
**How data was acquired**	The data were collected from the statistical offices of Botswana, Lesotho and South Africa.
**Data format**	Analysed
**Experimental factors**	Three datasets (for Lesotho, Botswana and South Africa) were combined in order to compare the level retail price differences over two specific periods.
**Experimental features**	The data were compared between the two periods which coincide with the periods before and after the introduction of macroeconomic policy shock in Botswana.
**Data source location**	Lesotho Bureau of Statistics, Botswana Central Statistical Office, Statistics South Africa.
**Data accessibility**	Data is available in this article.

**Value of the data**

• The data underscores the significance of analysing price disparities at a micro level across countries can unpack some important insights on heterogeneity in the level of product prices across cities, even within narrowly defined products.
• The described study is important for the analysis of product market integration across countries.
• The time-dimension of the data provide further information on how changes in transaction costs affect retail prices over time in the Southern African region.
• The availability of data allows for re-use and replicability of the results thereof.

## 1 Data, experimental design, materials and methods

### 1.1 Data

The data provided is based on retail product prices underlying the consumer price index (CPI) in Botswana, Lesotho and South Africa, provided at the product level and varies across cities and across time.

Each product has several price records and has information on the date (month and year), city, product and unit codes and units of measurements of that product.

The data presented in this article covers the two periods (June 2004- May 2006) and (January 2007 – December 2008) for each of the three countries.

### 1.2 Experimental design, materials and methods

The data represent a quasi-experiment of two large macroeconomic policy reforms that were introduced in Botswana in May 2005 and in January 2008 [1]. The design is such that the retail product prices were observed a year before and after the introduction of each of the policy reforms [2]. A very detailed concordance of these products describes names, units and in some cases, brands using the product lists obtained from the Botswana, Lesotho and SA statistical offices. A set of regions in the three countries where the price data for the selected products were available for all periods were then identified (Table 1). Finally, the data accounts for the differences in tax rates on goods and services (sales tax and Value added tax) and exchange rates between Botswana and Lesotho and South Africa.

**Table 1:** presents the descriptions of the retail products.

**Lesotho**	**Botswana**	**South Africa**	**South African sourced products**	**Product types**
Soft drink, Coca-Cola,340 ml can	Coca Cola, 340 ml can	Coca-Cola Soft Drink, 340 ml can	Yes	**
Candles, Newden, packet of six	Candles, packet of six	White Candles, packet of six	Yes	
Wine (Non-Sparkling), White JC Leroux,750 ml	Cane Spirit, (Mainstay), 750 ml	White Wine - South African, 750 ml	Yes	**
Women's footwear, Ladies’ dress shoes	Ladies flat working shoes, size 6	Court shoes - Genuine leather upper, pair	No	
Peanut Butter, Blackcat,410 g	Peanut Butter, 400 g	Peanut Butter, 410 g	Yes	**
Electric Kettle; material-plastic	Kettle (4 cups, not electric)	Kettle, each	Yes	
Cake flour, 2.5 kg	White bread flour, 2.5 kgs	Cake flour, 2.5 Kg	Yes	**
1Bed, Base and mattress	Double bed with mattress, (Sealy Posture)	Double Bed base with inner-spring Mattress	Yes	
Green beans, 500 g	Beans, 500 g	Beans, 500 g	No	*
Cabbage, 1 kg	Cabbage, 1 kg	Cabbage, 1 kg	No	*
Bread, white, one loaf	Bread, one white loaf, not sliced	Loaf of white bread, 700 g	No	*
Sugar, white, 2.5 kg	Sugar, white, 2 kg	Sugar, white, 2.5 kg	Yes	**
Peas, Koo canned,410 g	Tinned peas, 410 g	Peas, 410 g	Yes	**
Cereal, cornflakes,500 g	Corn Flakes, 500 g box (Kellogg's)	Cereal Flakes (e.g. Corn Flakes), 500 g	Yes	**
Biscuits, Marie blue label, 200 g	Biscuits, (Eet-Sum-More), 200 g	Marie Biscuits, 200 g	Yes	*
Oil, sunflower,750 ml	Sunflower cooking oil, 750 ml bottle	Sunflower oil, 750 ml	Yes	**
Macaroni, Fattis and Monis,500 g	Spaghetti, 500 g	Macaroni, 500 g	Yes	**
Jik, (bleach), 750 ml	Bleach, (JIK), 750 ml	Bleach, 750 ml	Yes	

1 Prices are collected from SA furniture chain stores *Perishables products **Non-perishable products.

* Non-perishable products.

** Perishables products.

Table A1, in the Appendix A, presents summary statistics (mean, median and standard deviation) of the natural log of the monthly retail price by product for each country. Table A2, Table A3 present the mean, median and standard deviation of log prices by month over the two periods for each of the three countries. Table A4, Table A5 presents the summary statistics on the mean absolute values in log differences between South Africa and Lesotho and between South Africa and Botswana for each product in the sample.

This data is important in facilitating analysis of prices at a unit level that enables an understanding of actual pricing conduct at the most basic level. This is important for academic advancement in building macroeconomic models that better incorporate the characteristics of economic agents at the micro level.

## Figures and Tables

**Table A1:** Log prices by products in the sample.

Product description	Botswana			Lesotho			South Africa		
	Simple average	Median	Standard deviation	Simple average	Median	Standard deviation	Simple average	Median	Standard deviation
**First period (June 2004 - May 2006)**									
Bed, Base and mattress	7.734	7.802	0.308	7.964	7.958	0.217	7.836	7.852	0.304
Biscuits, Marie blue label, 200 g	1.952	1.943	0.064	1.968	1.969	0.065	1.395	1.384	0.161
Bread, white, One loaf	1.443	1.434	0.081	1.2	1.219	0.121	1.517	1.545	0.128
Cabbage, 1 kg	2.152	2.154	0.102	2.117	2.138	0.09	2.117	2.099	0.121
Cakeflour, 2.5 kg	3.301	3.302	0.075	2.412	2.412	0.068	2.52	2.484	0.144
Candles, Newden, packet of six	1.709	1.703	0.124	1.500	1.501	0.113	1.709	1.641	0.222
Cereal, cornflakes, 500 g	2.93	2.928	0.105	2.648	2.671	0.162	2.691	2.707	0.124
Electric Kettle; material-plastic	3.608	3.610	0.144	3.565	3.525	0.257	4.793	4.787	0.237
Frozen green beans, 500 g	1.904	1.905	0.104	1.502	1.51	0.063	1.628	1.646	0.198
Jik, 750 ml	2.118	2.119	0.098	2.031	2.034	0.064	1.778	1.811	0.224
Macaroni, Fattis and Monis,500 g	1.69	1.696	0.078	1.695	1.696	0.058	1.635	1.620	0.130
Oil, Sunflower, 750 ml	2.162	2.167	0.076	1.993	1.990	0.067	1.918	1.886	0.123
Peanut butter, blackcat, 410 g	2.37	2.375	0.068	2.329	2.327	0.045	2.213	2.223	0.117
Peas, Koo canned, 410 g	1.974	1.966	0.071	1.413	1.379	0.164	1.770	1.829	0.214
Soft drink, Coca-Cola, 340 ml can	1.442	1.437	0.056	1.652	1.662	0.044	1.416	1.386	0.06
Sugar, white, 2.5 kg	2.686	2.68	0.062	2.515	2.510	0.051	2.559	2.587	0.083
Wine (Non-Spackling), White JC Leroux, 750 ml	3.716	3.746	0.206	2.592	2.565	0.207	2.904	2.913	0.239
Women's footwear, Ladies’ dress shoes	4.573	4.555	0.187	4.592	4.605	0.323	5.277	5.296	0.401

**Second period (January 2007 – December 2008)**									
Bed, Base and mattress	8.246	8.274	0.291	7.819	7.856	0.415	7.907	7.899	0.249
Biscuits, Marie blue label, 200 g	2.215	2.178	0.206	2.158	2.14	0.144	1.589	1.579	0.22
Bread, white, One loaf	1.809	1.766	0.242	1.517	1.458	0.136	1.774	1.754	0.197
Cabbage, 1 kg	2.336	2.322	0.163	2.146	2.165	0.125	2.224	2.216	0.129
Cakeflour,2.5 kg	3.538	3.498	0.228	2.667	2.611	0.218	2.700	2.649	0.252
Candles, Newden, packet of six	2.293	2.226	0.278	2.049	1.999	0.257	2.338	2.332	0.236
Cereal, cornflakes, 500 g	3.092	3.079	0.183	2.818	2.833	0.192	2.780	2.777	0.092
Electric Kettle; material-plastic	4.738	4.719	0.197	3.688	3.807	0.351	4.934	4.875	0.355
Frozen green beans, 500 g	2.411	2.398	0.184	2.230	2.247	0.128	2.034	2.014	0.198
Jik, 750 ml	2.089	2.059	0.194	1.949	1.935	0.158	1.907	1.862	0.201
Macaroni, Fattis and Monis, 500 g	2.668	2.567	0.300	2.386	2.250	0.358	2.352	2.267	0.343
Oil, Sunflower,750 ml	2.609	2.587	0.146	2.503	2.504	0.137	2.464	2.475	0.134
Peanut butter, blackcat, 410 g	2.161	2.151	0.150	1.602	1.568	0.278	1.930	1.982	0.24
Peas, Koo canned,410 g	1.629	1.609	0.105	1.771	1.777	0.084	1.455	1.448	0.056
Soft drink, Coca-Cola, 340 ml can	2.084	2.063	0.233	1.664	1.652	0.118	1.937	1.968	0.237
Sugar, white, 2.5 kg	2.892	2.877	0.112	2.708	2.71	0.145	2.693	2.687	0.08
Wine (Non-Spackling), White JC Leroux, 750 ml	2.834	2.822	0.147	3.038	2.867	0.502	3.296	3.296	0.227
Women's footwear, Ladies’ dress shoes	4.386	4.526	0.603	4.752	4.776	0.499	5.420	5.521	0.340

Notes: The data are extracted from the price database that is used to construct the Consumer price index (CPI) for each country and are consistent throughout the whole period.

**Table A2:** Log prices by across time for the period June 2004 – May 2006.

Month	Botswana			Lesotho			South africa		
	Simple average	Median	Standard deviation	Simple average	Median	Standard deviation	Simple average	Median	Standard deviation
May 2004	2.763	2.214	1.470	2.517	2.094	1.556	2.397	1.962	1.458
June 2004	2.756	2.209	1.471	2.529	2.114	1.592	2.430	1.986	1.518
July 2004	2.759	2.217	1.471	2.529	2.063	1.586	2.428	2.014	1.516
August 2004	2.787	2.246	1.473	2.506	2.073	1.566	2.428	2.006	1.520
September 2004	2.775	2.230	1.474	2.516	2.075	1.569	2.420	1.957	1.526
October 2004	2.768	2.216	1.477	2.517	2.068	1.562	2.410	1.949	1.517
November 2004	2.758	2.205	1.471	2.516	2.070	1.568	2.415	1.944	1.515
December 2004	2.760	2.210	1.472	2.524	2.071	1.567	2.425	1.949	1.523
January 2005	2.776	2.216	1.473	2.511	2.089	1.556	2.421	1.944	1.518
February 2005	2.767	2.213	1.472	2.485	2.061	1.582	2.426	1.987	1.519
March 2005	2.792	2.226	1.470	2.456	2.031	1.494	2.419	1.987	1.517
April 2005	2.787	2.280	1.473	2.508	2.063	1.580	2.416	2.010	1.511
May 2005	2.691	2.131	1.473	2.508	2.081	1.556	2.421	2.006	1.513
June 2005	2.705	2.137	1.475	2.524	2.080	1.553	2.422	2.027	1.515
July 2005	2.707	2.147	1.472	2.495	2.046	1.58	2.389	2.020	1.461
August 2005	2.707	2.135	1.470	2.491	2.059	1.543	2.383	2.027	1.456
September 2005	2.703	2.129	1.473	2.515	2.070	1.549	2.424	1.997	1.479
October 2005	2.719	2.146	1.473	2.495	2.048	1.559	2.418	2.027	1.464
November 2005	2.706	2.128	1.477	2.506	2.063	1.546	2.295	1.987	1.331
December 2005	2.702	2.107	1.495	2.513	2.088	1.545	2.287	2.000	1.326
January 2006	2.691	2.112	1.497	2.518	2.086	1.542	2.322	2.010	1.390
February 2006	2.698	2.117	1.499	2.525	2.090	1.557	2.329	2.006	1.387
March 2006	2.713	2.135	1.496	2.523	2.082	1.555	2.654	2.027	1.696
April 2006	2.720	2.146	1.501	2.518	2.076	1.560	2.665	1.964	1.736
May 2006	2.764	2.170	1.501	2.515	2.076	1.556	2.618	1.970	1.646
June 2006	2.788	2.191	1.502	2.518	2.051	1.560	2.605	1.963	1.634

Notes: The dates included in the sample are selected based on the two macroeconomic policy shocks in Botswana in 2005 and in 2008.

**Table A3:** Log prices by across time for the period January 2007 – December 2008.

Month	Botswana			Lesotho			South africa		
	Simple average	Median	Standard deviation	Simple average	Median	Standard deviation	Simple average	Median	Standard deviation
January 2007	2.861	2.330	1.537	2.712	2.172	1.641	2.647	2.038	1.631
February 2007	2.887	2.372	1.561	2.652	2.151	1.596	2.641	2.066	1.631
March 2007	2.897	2.378	1.561	2.646	2.138	1.588	2.652	2.073	1.608
April 2007	2.907	2.408	1.552	2.606	2.159	1.488	2.690	2.080	1.608
May 2007	2.904	2.403	1.553	2.636	2.176	1.528	2.734	2.102	1.671
June 2007	2.898	2.400	1.525	2.679	2.184	1.580	2.743	2.115	1.644
July 2007	2.916	2.407	1.575	2.668	2.180	1.535	2.732	2.161	1.598
August 2007	2.921	2.425	1.563	2.673	2.234	1.491	2.732	2.172	1.625
September 2007	2.914	2.429	1.563	2.697	2.262	1.504	2.763	2.196	1.578
October 2007	2.906	2.43	1.558	2.666	2.235	1.457	2.831	2.230	1.601
November 2007	2.932	2.457	1.554	2.721	2.242	1.505	2.874	2.286	1.623
December 2007	2.931	2.465	1.526	2.769	2.272	1.530	2.914	2.401	1.620
January 2008	2.992	2.541	1.522	2.747	2.300	1.464	2.849	2.291	1.583
February 2008	3.047	2.543	1.535	2.696	2.280	1.443	2.856	2.302	1.550
March 2008	3.077	2.608	1.503	2.782	2.307	1.488	2.910	2.397	1.540
April 2008	3.060	2.592	1.477	2.769	2.318	1.474	2.924	2.432	1.508
May 2008	3.074	2.630	1.465	2.802	2.351	1.436	2.996	2.482	1.550
June 2008	3.105	2.643	1.466	2.842	2.373	1.436	2.952	2.456	1.527
July 2008	3.104	2.644	1.473	2.898	2.451	1.436	3.010	2.484	1.527
August 2008	3.127	2.660	1.469	2.906	2.460	1.428	2.998	2.554	1.478
September 2008	3.163	2.687	1.472	2.910	2.464	1.416	3.014	2.624	1.451
October 2008	3.264	2.829	1.465	2.945	2.520	1.449	3.058	2.579	1.492
November 2008	3.279	2.877	1.457	2.967	2.579	1.448	3.072	2.624	1.494
December 2008	3.262	2.871	1.424	2.986	2.623	1.449	3.020	2.624	1.427

Notes: The dates included in the sample are selected based on the two macroeconomic policy shocks in Botswana in 2005 and in 2008.

**Table A4:** Mean absolute log price deviations by products for the period (May 2004-December 2008).

**Product description**	**June2004-May2006**		**Jan2007-Dec2008**	
	SA-LES	SA-BOTS	SA-LES	SA-BOTS
**Individual products**				
Bed, Base and mattress	0.326	0.366	0.420	0.438
Biscuits, MARIE blue label, 200 g	0.557	0.537	0.552	0.618
Bread, white, One loaf	0.361	0.139	0.272	0.144
Cabbage, 1 kg	0.110	0.122	0.149	0.159
Cakeflour,2.5 kg	0.148	0.759	0.086	0.822
Candles, Newden, packet of six	0.256	0.172	0.131	0.222
Cereal, cornflakes,500 g	0.167	0.240	0.161	0.318
Electric Kettle; material-plastic	1.206	1.219	1.239	0.339
Green beans, 500 g	0.273	0.347	0.217	0.379
Jik, 750 ml	0.122	0.117	0.105	0.190
Macaroni, Fattis and Monis,500 g	0.124	0.237	0.143	0.308
Oil, Sunflower,750 ml	0.142	0.169	0.088	0.153
Peanut butter, blackcat,410 g	0.424	0.203	0.384	0.243
Peas, KOO canned,410 g	0.233	0.061	0.270	0.121
Soft drink, Coca-Cola can,340 ml	0.201	0.289	0.320	0.216
Sugar, white,2.5 kg	0.081	0.142	0.087	0.202
Wine (Non-Sparkling), White JC Leroux,750 ml	0.359	0.805	0.458	0.451
Women's footwear, Ladies’ dress shoes	0.767	0.863	0.784	1.065

**Product groups**				
Alcoholic beverages	0.359	0.805	0.458	0.451
Non-alcoholic beverages	0.233	0.061	0.312	0.356
Food	0.219	0.275	0.219	0.305
Clothing and footwear	0.767	0.863	0.784	1.065
Household furniture and equipment	0.522	0.619	0.793	0.387
Household operations	0.266	0.271	0.188	0.327

Note: This table presents the summary statistics on the mean absolute values of the difference in log differences between South Africa and Lesotho and between South Africa and Botswana for each product in the sample. The data are presented for the year before and the year after an exchange rate policy shock in 2005 and a monetary policy shock in 2008, and are aggregated using the monthly price data for each city-pair.

**Table A5:** Mean absolute log price deviations over time (May 2004-December 2008).

**Date**	**May2004-April2006**		**January2007-December2008**		
	SA-LES	SA-BOTS	Month	SA-LES	SA-BOTS
May 2004	0.254	0.335	January 2007	0.339	0.391
June 2004	0.25	0.339	February 2007	0.316	0.389
July 2004	0.256	0.36	March 2007	0.305	0.389
August 2004	0.259	0.355	April 2007	0.299	0.386
September 2004	0.25	0.347	May 2007	0.321	0.38
October 2004	0.245	0.338	June 2007	0.314	0.368
November 2004	0.256	0.339	July 2007	0.308	0.357
December 2004	0.258	0.347	August 2007	0.314	0.376
January 2005	0.245	0.337	September 2007	0.321	0.37
February 2005	0.249	0.362	October 2007	0.327	0.363
March 2005	0.254	0.357	November 2007	0.313	0.367
April 2005	0.257	0.293	December 2007	0.316	0.343
May 2005	0.253	0.301	January 2008	0.302	0.342
June 2005	0.255	0.309	February 2008	0.311	0.33
July 2005	0.254	0.313	March 2008	0.313	0.337
August 2005	0.258	0.319	April 2008	0.311	0.318
September 2005	0.252	0.324	May 2008	0.32	0.325
October 2005	0.241	0.275	June 2008	0.293	0.328
November 2005	0.249	0.274	July 2008	0.321	0.326
December 2005	0.24	0.272	August 2008	0.299	0.304
January 2006	0.244	0.270	September 2008	0.293	0.325
February 2006	0.324	0.372	October 2008	0.306	0.389
March 2006	0.315	0.371	November 2008	0.309	0.386
April 2006	0.311	0.386	December 2008	0.313	0.379

Note: This table presents the summary statistics on the mean absolute value of the difference in log product prices between city pairs in South Africa and Lesotho and between city-pairs in South Africa and Botswana for each month over the periods May 2004 – April 2006 and January 2007 to December 2008.

## Appendix A

See Table A1, Table A2, Table A3, Table A4, Table A5.
